# RNA Elements in Open Reading Frames of the Bluetongue Virus Genome Are Essential for Virus Replication

**DOI:** 10.1371/journal.pone.0092377

**Published:** 2014-03-21

**Authors:** Femke Feenstra, René G. P. van Gennip, Sandra G. P. van de Water, Piet A. van Rijn

**Affiliations:** 1 Department of Virology, Central Veterinary Institute of Wageningen UR (CVI), Lelystad, The Netherlands; 2 Department of Infectious Diseases & Immunology, Faculty of Veterinary Medicine, Utrecht University, Utrecht, The Netherlands; 3 Department of Biochemistry, Centre for Human Metabonomics, North-West University, Potchefstroom, South Africa; The Pirbright Institute, United Kingdom

## Abstract

Members of the *Reoviridae* family are non-enveloped multi-layered viruses with a double stranded RNA genome consisting of 9 to 12 genome segments. Bluetongue virus is the prototype orbivirus (family *Reoviridae,* genus *Orbivirus*), causing disease in ruminants, and is spread by *Culicoides* biting midges. Obviously, several steps in the *Reoviridae* family replication cycle require virus specific as well as segment specific recognition by viral proteins, but detailed processes in these interactions are still barely understood. Recently, we have shown that expression of NS3 and NS3a proteins encoded by genome segment 10 of bluetongue virus is not essential for virus replication. This gave us the unique opportunity to investigate the role of RNA sequences in the segment 10 open reading frame in virus replication, independent of its protein products. Reverse genetics was used to generate virus mutants with deletions in the open reading frame of segment 10. Although virus with a deletion between both start codons was not viable, deletions throughout the rest of the open reading frame led to the rescue of replicating virus. However, all bluetongue virus deletion mutants without functional protein expression of segment 10 contained inserts of RNA sequences originating from several viral genome segments. Subsequent studies showed that these RNA inserts act as RNA elements, needed for rescue and replication of virus. Functionality of the inserts is orientation-dependent but is independent from the position in segment 10. This study clearly shows that RNA in the open reading frame of *Reoviridae* members does not only encode proteins, but is also essential for virus replication.

## Introduction

The family of *Reoviridae* consists of non-enveloped viruses with a multi-layered capsid. They have a double stranded RNA (dsRNA) genome, consisting of 9 to 12 genome segments, and one copy of each segment is efficiently recruited and incorporated into each virus particle [1].

Bluetongue virus (BTV, genus: *Orbivirus*) is one of the most extensively studied *Reoviridae* members, and is transmitted to ruminants by *Culicoides* biting midges. Clinical manifestations associated with BTV infections can course from subclinical to severe haemorrhagic disease, characterized by fever, lameness, coronitis and swelling of the head, particularly the lips and tongue [2], [3]. Bluetongue (BT) is endemic in many tropical and subtropical regions and in some regions with a temperate climate, including large parts of the Americas, Africa, southern Asia and northern Australia [4]. There are at least 26 different BTV serotypes identified [5], [6], [7].

BTV virions (∼80 nm) consist of seven structural proteins (VP1 - VP7) forming an architecturally complex structure of an inner (VP3), middle (VP7) and outer (VP2 and VP5) capsid layer. These layers encapsidate the viral polymerase (VP1) [8], capping enzyme (VP4) [9] and helicase (VP6) [10], as well as the 10 dsRNA genome segments (Seg-1 - Seg-10) [2]. In addition, the BTV genome encodes four non-structural proteins (NS1 - NS4) [11], [12]. It is unknown how the RNA segments are exactly located within the virion. Most likely, these are highly ordered, in which several structural proteins (VP1, VP3, VP4, VP6), known for their ability to bind RNA, might be involved [8], [13], [14].

For successful virus replication, RNA segments are specifically recognized by viral proteins at different stages in the replication cycle, such as transcription, extrusion from core particles, translation, recruitment into viral inclusion bodies (VIBs), replication and assembly of new virus particles. The mechanism for selective packaging of the genome segments is still one of the most prominent and intriguing questions in this research field.

In orbivirus replication, after cell entry and removal of the outer shell, core particles transcribe capped mRNAs originating from all viral segments, which are extruded into the cytoplasm. These mRNAs are recruited from the cytoplasm into VIBs formed by NS2. NS2 may has a role in the recruitment of RNA from the cytoplasm by binding to the 5′- and 3′-untranslated regions (UTRs). However, undefined RNA sequences in the open reading frame (ORF) are also recognized by NS2 [15], [16]. Since dsRNA is only associated with virus particles, the recruitment of RNA likely occurs at the single stranded RNA level [17]. NS1 specifically enhances translation of viral mRNAs in the cytoplasm, likely by specific recognition of viral 3′-end sequences [18]. For mammalian orthoreoviruses, recognition signals for packaging in the 5′UTR have previously been identified [19], whereas for orbiviruses these recognition signals are mainly unknown. Since UTRs and, especially the 5′-UTRs, of BTV segments are extremely short (6-59 nucleotides) [20], and since RNA-protein interactions are important in numerous replication events, it is likely that coding sequences adjacent to the UTRs are also involved in recognition by proteins. Viral proteins have previously been recognized for their ability to specifically bind coding RNA [21], [22]. For orbiviruses such recognition sequences in coding regions have not been identified.

Until recently, research on sequences in coding *Reoviridae* RNA important for virus replication was hampered by the dual function of this RNA in both translation and replication. Reverse genetics has been developed for several BTV strains [23], [24] and mutants and reassortants of BTV have been generated [12], [25], [26], [27], [28]. BTV Seg-10 protein products NS3/NS3a were assumed to be essential for virus growth [25], [26], but we have recently demonstrated that NS3/NS3a expression is not required for *in vitro* propagation of BTV [29]. NS3/NS3a are membrane proteins involved in virus release and IFN antagonism [25], [26], [30], [31]. BTV without protein expression from Seg-10 enabled us to study the function of coding RNA in virus replication. In the present study, we show that RNA sequences in the BTV ORF are essential for virus replication, and that these RNA sequences can be complemented *in cis* by RNA inserts from several other genome segments. These findings are a first step to define RNA sequences involved in replication of *Reoviridae* members.

## Materials and Methods

### Cell culturing

BSR cells (a clone of baby hamster kidney (BHK) cells [32]) were kindly provided by Polly Roy (London School of Hygiene and Tropical Medicine) and maintained in Dulbecco’s modified Eagle’s medium (DMEM, Invitrogen) supplemented with 5% fetal bovine serum (FBS), 100 IU/ml penicillin/streptomycin (Gibco) and 2.5 μg/ml fungizone (Gibco).

### Plasmids with cDNAs of genome segments

**Figure 1:**
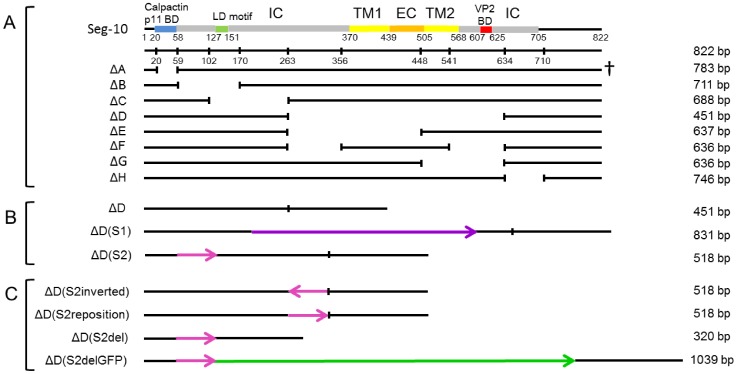
Deletion mutant Seg-10 used in reverse genetics for virus rescue. Deletions were made throughout the ORF of Seg-10. Mutant ΔA was not viable as indicated by a cross. Protein domains encoded by Seg-10 are indicated using different colours. BD = binding domain, LD = late domain, IC = intracellular, TM = trans membrane, EC = extracellular. Nucleotide positions are indicated with numbers. Segment length is indicated next to the illustrations. (A) Mutant segments with consecutive deletions throughout the original Seg-10. (B) Mutants based on segment ΔD, but with inserted viral sequences. Insertions of Seg-1 and Seg-2 are shown in purple and pink, respectively. The orientation of insertions are indicated by arrows. (C) ΔD(S2) segments, but with the insertion in a different location or orientation or with an additional deletion or with the GFP sequence(bright green) inserted.

Plasmids containing cDNA of Seg-1 to Seg-10 of BTV1 (Genbank accession numbers FJ969719-FJ969728) and Seg-10 of BTV8 (AM498060) have been described [23], [24]. Plasmids with mutated cDNA of Seg-10 were constructed by deletion or replacement of regions in the ORF (Figure 1) by standard cloning procedures using restriction enzymes or were synthesized by Genscript Corporation (Piscataway NJ, USA). Seg-10 with deletion ΔC was made using restriction enzymes BsaAI and BsmBI, ΔD using BsmBI and PsiI and ΔH using PsiI and Bsu36I (New England Biolabs). ΔD(S2)del had an additional NcoI-PsiI deletion. All other deletion Seg-10 mutants were synthetically generated. Only deletion ΔF and ΔG did not disturb the reading frame. Plasmids were transformed and maintained in DH5α *E.coli* competent cells (Invitrogen) and were isolated using the High Pure Plasmid Isolation Kit (Roche) or the QIAfilter Plasmid Midi Kit (Qiagen).

### *In vitro* RNA transcription

Plasmid DNA was linearized by restriction enzymes as described earlier [23] and purified using standard phenol-chloroform extraction. One μg linear DNA was used as a template for *in vitro* RNA transcription using the MESSAGE mMACHINE T7 Ultra Kit (Ambion) as described previously [23]. Synthesized capped RNA molecules were purified using the MEGAclear kit (Ambion), according to the manufacturer’s protocol and were stored at -80°C.

### Rescue of BTV with mutated genome segment 10

BTV mutants were generated using reverse genetics as previously described [23]. In short, 10^5^ BSR cells were transfected in a 24-wells plate using 1.5 μl lipofectamin 2000 (Invitrogen) and 600 ng RNA in total, containing Seg-1, 3, 4, 5, 8, and 9 in equimolar amounts, encoding VP1, VP3, VP4, NS1, NS2 and VP6 respectively. Eighteen hours post transfection, BSR monolayers were transfected again with in total 600 ng of all 10 RNA segments in equimolar amounts. All transfections were performed in duplicate. Wells were screened for cytopathogenic effect (CPE) at 48 h post transfection and one well was fixed with methanol:aceton and immunostained with α-VP7 monoclonal antibody (MAb) (American Type Culture Collection (ATCC)-CRL-1875) according to standard procedures [33]. When no CPE or stained plaques were visible as a sign of virus replication, the duplicate well was passaged to be able to rescue mutants with delayed growth characteristics. Passaging of transfected cells was repeated, depending on the presence or absence of visible CPE or immunostained plaques. If transient VP7 expression was no longer detectable, the attempt to generate mutant BTV was considered as unsuccessful. Attempts were repeated at least two times to consider a certain mutation lethal. Transfected monolayers were passaged until at least 50% of the cells either showed CPE or were positive in immunostaining. Subsequently, BTV mutants were harvested by freeze thawing twice at –80°C. Then, fresh BSR monolayers were infected with these harvested cells in order to conclude that virus rescue was successful. Fresh BSR monolayers were infected three subsequent times to prepare virus stocks and to examine genetic stability of Seg-10.

### Sequencing of Seg-10 of BTV mutants

Viral RNA was isolated from 200 μl of infected cell culture medium using the High Pure Viral RNA kit (Roche) according to manufacturer’s protocol and eluted in 50 μl RNase-free water. Entire BTV Seg-10 was reverse transcribed and amplified using primers F-full-S10* (5′-GTTAAAAAGTGTCGCTGCC-3′) and R-full-S10 (5′-GTAAGTGTGTAGTGTCGCGCAC-3′) and the one-step RT-PCR kit (Qiagen). Briefly, 5 μl isolated RNA was added to 10 μl 5x Qiagen one-step PCR buffer, 2 μl dNTP mix, 0.6 mM of each primer and 2 μl enzyme mix in a total volume of 50 μl. Reverse transcription was performed for 30 min at 45°C. After an activation step of 15 min at 94°C, cDNA was amplified in 40 cycles of 1 min at 94°C, 1 min at 45°C and 2 min at 72°C, followed by a terminal extension step at 72°C for 10 min.

The amplicon was separated on a 1% agarose gel by electrophoresis and isolated using the Zymoclean gel DNA recovery kit (Zymo Research) according to the manufacturer′s protocol. The sequence of amplicons was determined using appropriate primers and the BigDye Terminator v1.1 Cycle Sequencing Kit (Applied Biosystems) in an ABI PRISMH 3130 Genetic Analyzer (Applied Biosystems). The complete consensus sequence was assembled and determined using Lasergene SeqMan Pro Software (DNASTAR, version 7.2.1).

### Growth curves of BTV mutants on BSR cells

BSR cells in wells of a 24-wells plate were infected with a multiplicity of infection (MOI) of 0.1. Virus was attached to the cells for 1.5 h at 37°C. By washing with medium, free circulating virus was removed and fresh medium was added. This time point was set as time point 0 (0 hours post infection, hpi). Incubation at 37°C was continued and supernatant from one of the wells was each time harvested at indicated time points between 0–54 hpi. An equal volume of fresh medium was added to the attached cells in the well of which the supernatant was harvested and virus in the cell fraction was harvested at the same time points after freeze thawing that well at –80°C. Virus titers of cell fractions and supernatants were determined by end point dilution on BSR cells and expressed as tissue culture infectious dose per ml (TCID_50_/ml). Experiments were independently repeated four times and significant differences in virus titers were determined using a paired Student’s T-test, with p<0.05.

### Analysis of dsRNA of BTV mutants by polyacrylamide gel electrophoresis

BSR monolayers were infected with mutant BTV. Medium was discarded at 24 hpi and 0.1 ml/cm^2^ Trizol was added to the cells and incubated for 5 min at room temperature. After harvesting, 0.2 ml chloroform/ml Trizol was added and the mixture was centrifuged for 10 min at 6,000 rpm. The water phase was isolated and 0.8 ml isopropanol/ml was added. Precipitated RNA was centrifuged for 30 min at 4°C and 13,000 rpm. The pellet was washed with 70% ethanol and dissolved in 100 μl RNase-free water. Fifty μl of 7M LiCl was added, followed by incubation for 30 min at –20°C to precipitate ssRNA. After centrifugation for 15 min at 4°C and 13,000 rpm, dsRNA was purified from the supernatant using the RNA clean and concentrator^tm^-5 kit (Zymo research) according to manufacturer’s protocol. Approximately 200 ng dsRNA was separated by 4–12% polyacrylamide gel electrophoresis (PAGE) and visualized by silver staining using the SilverXpress kit (Invitrogen).

## Results

### Deletions in the ORF encoding NS3a do not prevent virus rescue

**Figure 2:**
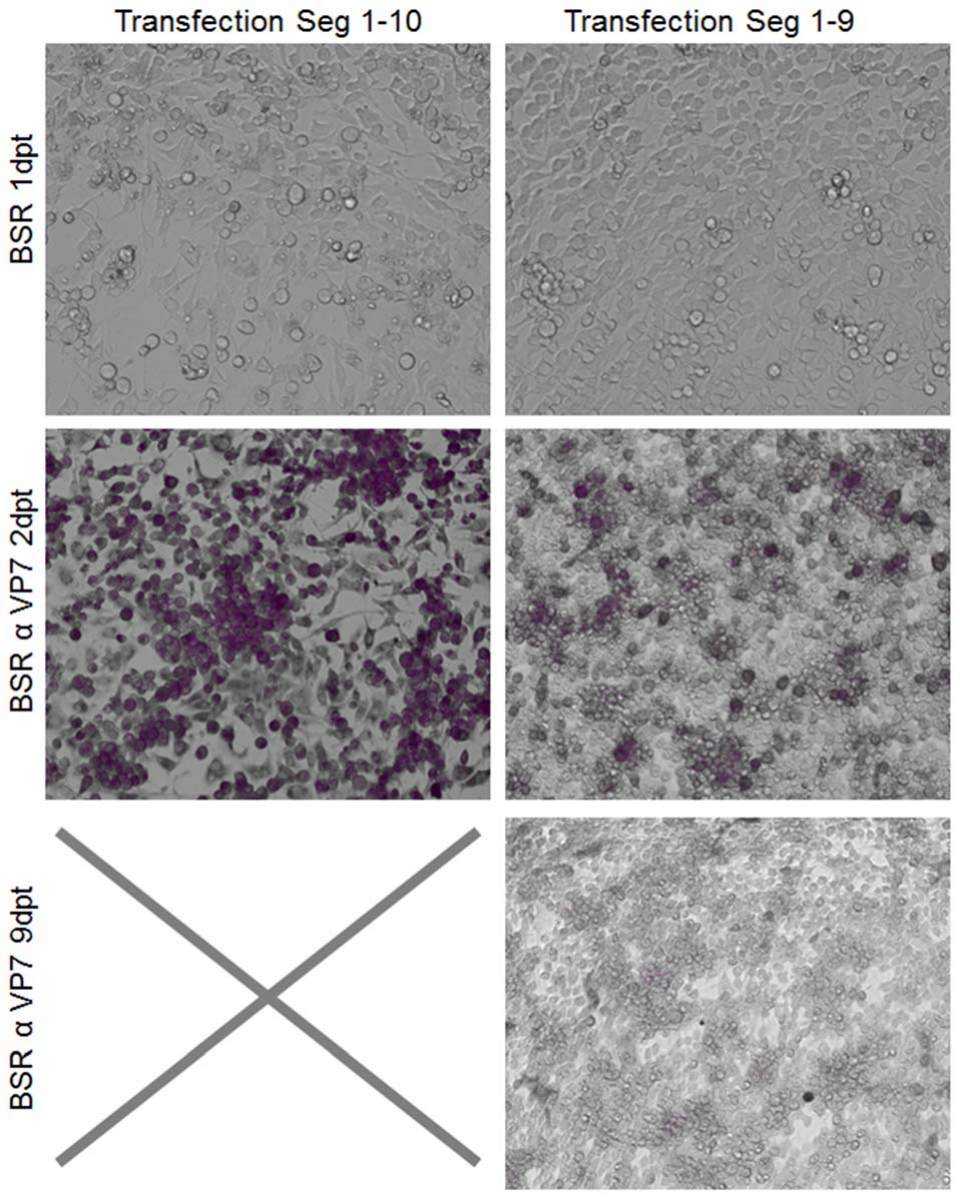
Seg-10 is essential for virus generation using reverse genetics. Transfected BSR cells 1dpt. CPE is visible in cells transfected with all ten BTV1 segments, whereas no CPE is observed in cells transfected with genome segments 1–9. At 2dpt almost all cells transfected with all ten BTV1 segments were immunostained with α VP7 MAb, whereas cells transfected with segments 1–9 showed transient expression only. Cells were passaged and stained at 9dpt. Complete CPE was observed for cells transfected with all ten segments. Therefore all cells died and could not be stained anymore, as indicated by a cross. At 9dpt no transient expression was detected in the cells transfected with 9 segments.

**Figure 3:**
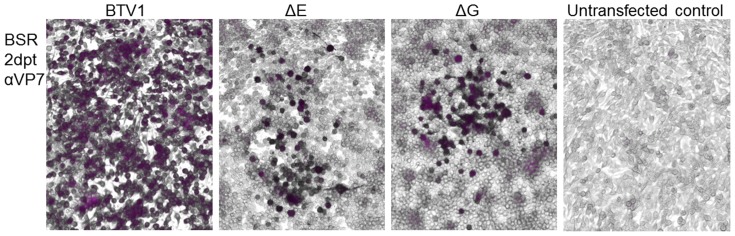
Representative result of rescue of mutant BTV with a deletion in Seg-10. BSR cells transfected with all segments of BTV1, BTV1 with Seg-10 ΔE or ΔG and untransfected control 2dpt stained with αVP7 MAb. Almost all cells transfected with the BTV1 segments were infected as was shown by immunostaining in purple. Smaller plaques of positive cells were visible in transfections with mutant Seg-10.

Previously, we have shown that gene products NS3 and NS3a encoded by Seg-10 are not essential for virus replication [29]. Firstly, we here confirmed that Seg-10 RNA is essential for generating BTV from *in vitro* synthesized RNAs using reverse genetics (Figure 2). Then, small deletions throughout Seg-10 were made, but deletions in the 5′- and 3′- UTRs were not included in this study as these are considered essential for virus generation using reverse genetics (Figure 1A). BTV deletion mutants were generated using reverse genetics, however, passaging of transfected cells was often needed to recover mutant BTV. Furthermore, immunostaining of transfected cells was needed to monitor recovery of virus, since most mutant BTVs did not show obvious CPE. Virus mutants with all intended deletions were generated, except for mutation ΔA. Apparently, the RNA sequence between both start codons in Seg-10 is essential for BTV generation. Rescue of BTV mutants for a set of small deletions throughout the ORF of NS3a was successful. Representative results of virus rescue with deletions in the ORF of NS3a are shown for deletion mutants ΔE and ΔG in Figure 3.

### BTV deletion mutants contain RNA inserts in Seg-10 from other genome segments

**Figure 4:**
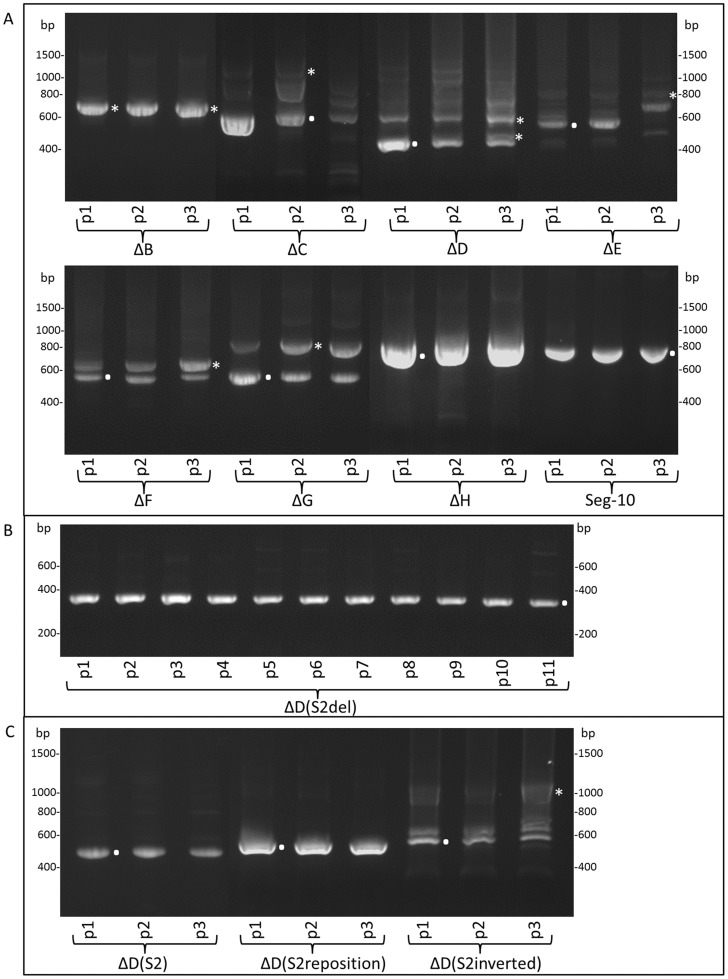
Stability of Seg-10 mutant viruses. (A) Stability of all Seg-10 deletion mutants was examined during three successive passages. Complete Seg-10 was amplified by RT-PCR, and Seg-10 stability was examined by gel electrophoresis. wtBTV1 was used as control. (B) Stability of Seg-10 of mutant virus ΔD(S2del) was confirmed for more than ten passages, by complete Seg-10 amplification using RT-PCR, gel electrophoresis and sequencing. (C) Stability of variants of Seg-10 mutant viruses with Seg-2 insertion during three successive passages. Seg-10 of ΔD(S2) and ΔD(S2reposition) were stable during three passages, whereas Seg-10 of ΔD(S2inverted) was not. Amplicons of the original Seg-10 mutant and Seg-10 mutant with additional inserted viral sequences are indicated by a dot and asterisks, respectively.

Deletions in Seg-10 of BTV mutants were confirmed by amplification of entire Seg-10 followed by sequencing. After three consecutive virus passages, Seg-10 was amplified, but cDNAs were larger than the expected size based on the respective deletions, as was examined by gel electrophoresis of RT-PCR products, except for mutants ΔB and ΔH. Subsequently, Seg-10 of each passage was amplified and subjected to agarose gel electrophoresis (Figure 4a). Seg-10 of BTV mutants ΔB and ΔH appeared stable for three passages, whereas all other studied BTV mutants showed larger amplicons than the expected size in the later passages, but often also already in the first passage. The original deletion Seg-10 of each BTV mutant could still be identified. However, BTV mutants contained several larger amplicons, indicating that there are virus subpopulations present containing Seg-10 different from the original deletion Seg-10, which quickly overgrew the original mutant. Since the larger amplicons are often already present in the first virus passage, they are apparently already present after only a few replication cycles of the intended deletion mutant virus.

**Figure 5:**
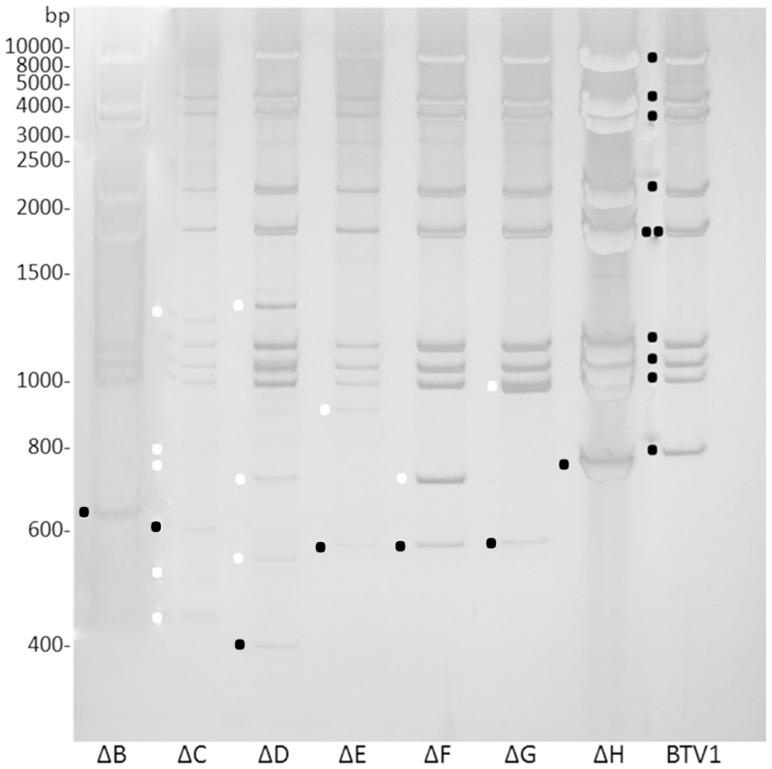
dsRNA of Seg-10 deletion mutant viruses. dsRNA was isolated from cells infected with passage 4 of all Seg-10 deletion mutant viruses. Black dots indicate the segments 1–10 of BTV1, with Seg-5 and Seg-6 almost at the same position in the gel. A black dot also indicates the band with the expected size of Seg-10 based on the deletion, for the different mutant viruses. White dots indicate Seg-10 bands of mutant viruses, different from deletion Seg-10 of the expected size. All mutant viruses contain a band with the size of the original deletion Seg-10. All Seg-10 deletion mutant viruses contain Seg-10 variants, except for ΔB and ΔH. Note that the ladder used is made of dsDNA, so the height in the gel of the dsRNA cannot be used to determine the exact size of the band.

dsRNA of BTV mutants of an additional passage on BSR cells clearly showed that Seg-1 to Seg-9 are identical in size to those of BTV1, but Seg-10 is not (Figure 5). In agreement with RT-PCR amplification results (Figure 4a), only BTV mutants ΔB and ΔH did not contain subpopulations of Seg-10. Note that RT-PCR amplification and dsRNA isolation is not completely comparable due to possible preferential amplification by RT-PCR and use of different virus passages. We conclude that deletion of several regions in Seg-10 resulted in genetic unstable but viable mutant BTVs.

Larger than expected Seg-10 amplicons indicated an insertion or duplication of RNA sequences. All designed deletions in Seg-10 were confirmed, but for each deletion mutant the sequence of at least one subpopulation with an insertion was also confirmed. All RNA inserts were from viral origin and were inserted in the positive orientation. However, inserts varied in length and originated from several genome segments. We found RNA inserts from genome segments 1, 2, 6, 8, 9 and a partial duplication of remaining sequences in deletion Seg-10. Further, these viral RNA sequences seemed to be randomly inserted, since inserts were found at different positions in deletion Seg-10 (Table 1). Inserted sequences matched with the respective original segment by MegAlign (DNA star, Lasergene, version 7.2.1) or Nblast (NCBI) (not shown). The insertions did not lead to recovery of NS3/NS3a protein expression. To examine possible similarities in RNA structure, RNA sequences were subjected to RNA structure predictions using Cylofold (http://cylofold.abcc.ncifcrf.gov/), RNASAlign (http://www.bio8.cs.hku.hk/RNASAlign/) and Alifold (http://rna.tbi.univie.ac.at/cgi-bin/RNAalifold.cgi) software. No obvious similarities in RNA structures were found, although many RNA structures could be predicted in all RNA inserts, and even the RNA insert of 67 base pairs originating from Seg-2 in ΔD contained a predicted RNA (pseudoknot) structure (not shown). We suggest that these inserts complement *in cis* for the deleted RNA sequence in Seg-10 by a yet unknown mechanism.

**Table 1:** Overview of Seg-10 deletion mutants with insertions.

Mutant Seg-10	Stability	Insertions	Position of the insert in Seg-10
ΔA*	-	-	-
ΔB	No	Insertion of one adenine	61
ΔC	No	Seg-9 (45–627)	280
ΔD	No	Seg-1 (333–712), Seg-1 (552–892), Seg-2 (770–836), Seg-8 (384–796)	193, 96, 60, 194
ΔE	No	Seg-1 (1278–1543), Seg-2 (780–835)	58, 58 (after the Seg-1 insert)
ΔF	No	Seg-10 duplication (453–540)	453
ΔG	No	Seg-1 (1187–1557)	441
ΔH	At least 3 passages	No additional modifications	-
ΔD(S2)	At least 3 passages	No additional modifications	-
ΔD(S2del)	At least 11 passages	No additional modifications	-
ΔD(S2reposition)	At least 3 passages	No additional modifications	-
ΔD(S2 inverted)	No	Seg-6 (bp 467–693)	633
ΔD(S2delGFP)	No	Seg-6 (bp 863–1059)	41

Stability of Seg-10 deletion mutants during virus growth is indicated. For unstable mutants, changes in Seg-10 are indicated and specified for segment number of origin and nucleotide numbering (between brackets) of the respective segment. The location of the insertion is indicated by the nucleotide number of full length Seg-10.

* BTV mutant with the ΔA deletion in Seg-10 was not viable.

### Viral *in cis* RNA elements are essential for virus rescue

We clearly showed that rescue of BTV with deletion Seg-10 results in virus mutants with additional RNA inserts. Two BTV mutants, ΔD(S2) with an insertion from Seg-2 (770–836, Table 1), and ΔD(S1) with an insertion from Seg-1 (333–712, Table 1) (Figure 1B), were directly reproduced using reverse genetics. Thus, cDNA of Seg-10 of ΔD(S1) and ΔD(S2) with the Seg-1 or Seg-2 insertion already present were used for *in vitro* RNA synthesis and subsequently used for virus rescue. Two days post second transfection (dpt), plaques were already clearly visible by immunostaining (not shown). Since ΔD mutant production was less efficient, this demonstrates that inserts of viral sequences in deletion Seg-10 increase the efficiency of virus rescue. Furthermore, except for ΔH (see discussion), deletion BTV mutants without quickly arising subpopulations containing inserts in Seg-10 could not be propagated, indicating that inserting these RNA inserts is essential for virus rescue. Genetic stability of newly rescued ΔD(S2) was confirmed for at least three virus passages by dsRNA analysis (not shown), and RT-PCR amplification of Seg-10 (Figure 4C).

Seg-10 of deletion BTV mutant ΔD(S2) was further shortened resulting in a Seg-10 of 320 base pairs in length, named ΔD(S2del) (Figure 1C). BTV mutant ΔD(S2del) was efficiently rescued without additional passages. Genetic stability of ΔD(S2del) was confirmed by 11 consecutive virus passages (Figure 4B). This demonstrates that the large deletion in Seg-10 can be complemented *in cis* by the Seg-2 sequence of only 67 base pairs in length.

The same Seg-2 insert was further analysed. First, the Seg-2 insert was repositioned further downstream in the cDNA of ΔD, named ΔD(S2reposition) (Figure 1C). Second, the Seg-2 insert in ΔD(S2reposition) was inverted (negative orientation) resulting in ΔD(S2inverted) (Figure 1C). BTV mutant ΔD(S2reposition) was rescued and appeared to be stable for at least three passages, whereas for ΔD(S2inverted) subpopulations of Seg-10 arose after one cell passage (Figure 4C). Apparently, the Seg-2 insert in the inverted orientation remained present, but was not functional in *in cis* complementation and advantageous sequences were quickly inserted similar to virus rescue for other deletions in Seg-10. These results show that the position of the viral insert is not important, whereas the orientation of the insert is crucial for its function in virus replication. We conclude that inserted RNA sequences are *in cis* RNA elements needed for virus replication and that these elements can originate from several genome segments.

**Figure 6:**
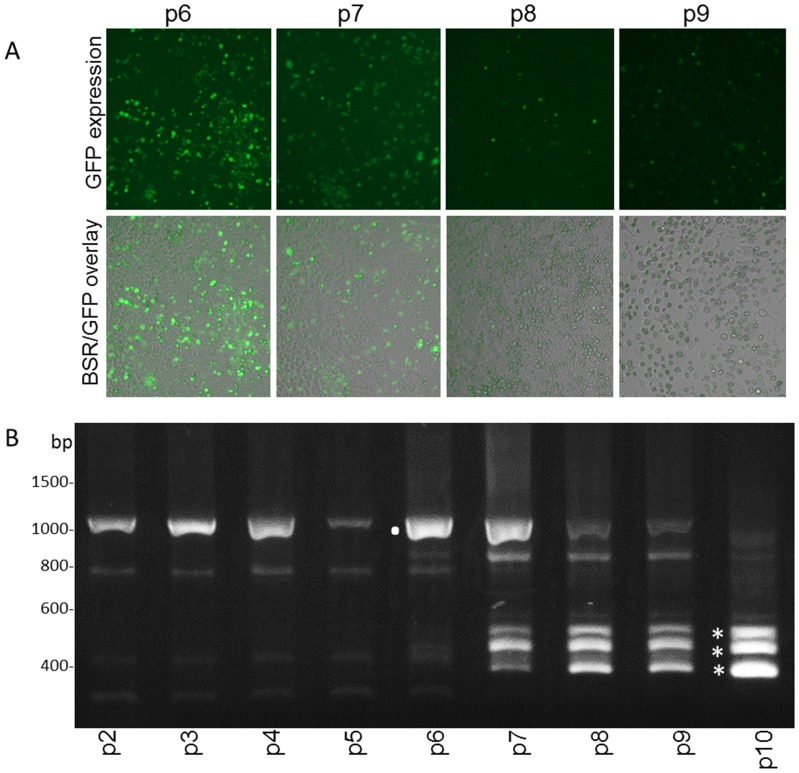
Stability of ΔD(S2delGFP) mutant virus. (A) ΔD(S2del) virus with the GFP sequence inserted (ΔD(S2delGFP)) was generated. GFP expression was obvious during several successive virus passages in BSR cells, as shown for passage 6 and 7 (p6, p7). GFP expression was less obvious after subsequent passages, as shown for passages 8 and 9 (p8, p9). (B) Genetic stability of Seg-10 of ΔD(S2delGPF) during ten passages was studied by RT-PCR amplification of Seg-10. The original Seg-10 of ΔD(S2delGFP) mutant virus was identified (.), but in subsequent passages additional smaller amplicons became more prominent (*). The middle small band has a deletion in the GFP sequence, the smallest amplicon has a larger deletion in the GFP sequence, and in the largest of the small amplicons, the Seg-2 insertion is also deleted, but a Seg-6 sequence is inserted instead.

The large deletion in ΔD(S2del) might enable insertion of non-viral sequences. The ORF of green fluorescent protein (GFP) was therefore inserted (in frame) downstream of the Seg-2 element in the cDNA of ΔD(S2del) (Figure 1C). Mutant BTV expressing GFP, ΔD(S2delGFP), was generated and GFP expression was clearly visible in seven consecutive virus passages on BSR cells (Figure 6A). Then, subsequent virus passages showed a drastic decrease in GFP expression. Indeed, RT-PCR amplification of Seg-10 showed instability of ΔD(S2delGFP) after about six passages(Figure 6B). This relatively long period of Seg-10 stability again shows the benefit of the presence of the Seg-2 sequence. RT PCR showed that in the thickest of three bands appearing in the sixth passage, part of the GFP sequence was deleted, whereas the Seg-2 insert was steady present. A subpopulation with even larger deletions in the ORF of GFP was also detected, and it seemed that this population had an advantage over the other subpopulations, since it is the thickest band in the 11^th^ passage. A subpopulation missing both a large part of GFP and the Seg-2 element was also identified. However, here the Seg-2 element was replaced by insertion of a Seg-6 RNA element. Again, Seg-10 subpopulations without additional viral sequence were not found, which strongly indicates that RNA elements from viral origin are essential for BTV replication. Further, foreign RNA sequences, such as the ORF of GFP, cannot compensate for deletions in Seg-10. We conclude that several RNA regions in the ORF encoding NS3a are needed for virus replication. Although the mechanism of this is unknown yet, we further conclude that the function of these RNA sequences can be complemented *in cis* by inserting RNA sequences of other genome segments in the sense orientation.

### Phenotype of deletion BTV mutants

**Figure 7:**
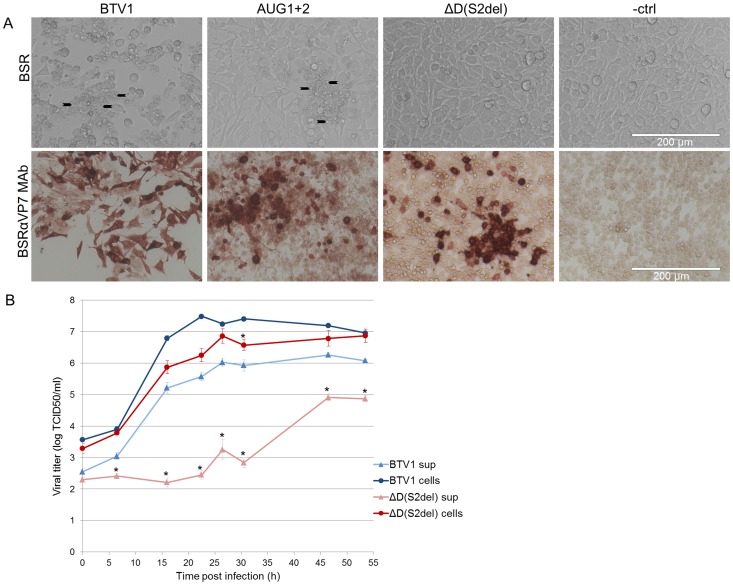
Phenotype and growth of wild type, AUG1+2 and ΔD(S2)del virus on BSR cells. (A) BSR cells, 1dpi, infected with MOI 0.1. CPE is clearly visible in BSR cells infected with BTV1. Upper row: Typical BTV1 CPE is indicated (arrows). Cells infected with the double ATG mutant (AUG1+2) also show CPE, but delayed. The ΔD(S2del) virus shows no CPE and infected cells look comparable to uninfected cells. Lower row: Infected monolayers were immunostained with αVP7 MAb. For BTV1 all cells are positive, AUG1+2 shows less positive cells and ΔD(S2del) only shows immunostaining of single cells or small groups of cells. (B) Virus titers of infected cells were examined in medium and cell fractions at time points up to 54 hpi. Virus titers in cell fractions are not significantly different for both viruses, except for 22 hpi. However, virus release in medium is significantly delayed and reduced for ΔD(S2del) virus compared to BTV1. Error bars represent SEM and asterisks indicate a significant difference in virus titer between ΔD(S2del) virus compared to BTV1 with p<0.05.

Our group showed that BTV mutants without NS3/NS3a expression (AUG1+2 mut) show reduced CPE and reduced release of virus in culture medium [29]. Unexpectedly, BTV mutant ΔD(S2del) caused CPE even less prominent than the previously described NS3/NS3a knockout BTV mutants. This might be due to the deletion of RNA, that was still present in the ATG1+2 mut virus or due to possible protein expression by the ATG1+2 mut virus, prohibited in the deletion mutant. However, virus replication is clearly visible by immunostaining of infected cells (Figure 7A). Growth curves of BTV1 and mutant ΔD(S2del) on BSR cells showed that virus replication in infected cells is slightly reduced, whereas release of ΔD(S2del) was more than 20 h delayed with respect to BTV1 and reached less high titers (Figure 7B).

## Discussion

The *Reoviridae* genome is composed of 9-12 linear dsRNA genome segments. Single copies of each genome segment need to be incorporated in the virus particle to form infectious virus. The exact mechanism by which this is orchestrated is unknown. Protein-RNA interactions specific for the virus segments in general and specific for each individual segment play an important role to direct this process in an efficient way. For BTV as representative of the orbivirus genus, proteins VP1, VP3, VP4, VP6, NS1, and NS2 have RNA binding capacity, but the exact RNA sequences involved in binding and segment recognition have not been elucidated [8], [34], [35], [36], [37], [38], [39], [40].

Studies on RNA binding in coding regions have been limited to cell-free systems, due to interference of introduced mutations with translation of supposed essential viral proteins. We have recently found that translation of NS3/NS3a from BTV Seg-10 is not essential for BTV replication [29]. This finding was a unique chance to study the role of RNA sequences in virus replication, irrespective of translation.

Virus rescue without adding RNA of Seg-10 using reverse genetics has failed, indicating that sequences in Seg-10 are truly essential for virus rescue, as has been shown for Seg-9 [27]. Indeed, formation of virus particles lacking one or more genome segments, as possible for members of other virus families such as *Bunyaviridae* [41], is not described for *Reoviridae*. BTV mutants with deletions in Seg-10 were generated, but deletion of RNA sequences between both start codons, in fact the 5′-UTR of NS3a (mutant ΔA), appeared detrimental for virus rescue, showing its importance. This sequence is highly conserved as is shown by its use in molecular diagnostics [42], [43], [44], difficulty to introduce point mutations in this region [45] and unsuccessful attempts by our group to generate mutant BTV with only eight silent mutations in this region [46].

Rescue of all other deletion mutants in the Seg-10 ORF was possible, but RNA inserts from several genome segments were found in deletion Seg-10, very quickly after virus rescue. The original deletion Seg-10 was still detectable, but detection was rapidly declining, whereas detection of Seg-10 with an insertion was rising. Although there is apparently still a small subpopulation in the virus pool present that does not contain the insertion, the original deletion mutant can never form a virus pool not containing subpopulations with insertions. This shows that the RNA inserts are essential for virus replication, since no virus pools without insertions can be generated.

The only mutant without additional insert was ΔH. BTV ΔH still expresses C-terminal truncated NS3/NS3a, as was confirmed by IPMA (not shown). C-terminal truncated NS3/NS3a is still functional [25], which is here confirmed by CPE induced by BTV ΔH in BSR cells. RNA inserts in Seg-10 of this mutant would lead to loss of NS3/NS3a functions and this loss is likely the cause that insertions were not found for mutant ΔH. In Seg-10 of mutant virus ΔB, an insertion of only one adenine upstream from the second start codon was identified. This insertion restored the reading frame of NS3, and resulted in expression of 178 N-terminal amino acids of NS3. This insertion was likely selected because of the recovery of expression of truncated NS3, and not because of *in cis* complementation. Again, like for ΔH, this confirms that non-essential NS3 is highly beneficial for BTV replication.

The RNA sequences were probably inserted by replicative recombination events. Such events are common in viral evolution [47], [48], [49]. dsRNA segments of bacteriophage Φ6 have also shown inserts after changing the sequence of one segment [50]. Intersegment recombination in rotavirus [51], but also in orbivirus [52], has been suggested based on sequence analyses and differences in homology between regions within segments. Since intersegmental recombination in wild type virus will disturb expression of functional proteins, such events are lethal or disadvantageous in virus replication. However, in our experiments, disturbance of functional NS3/NS3a protein expression was already induced by the deletions made in the open reading frame, and is not lethal. Recombination events in deletion Seg-10 did therefore not further disturb translation of NS3/NS3a, and are highly favourable for virus replication as was shown by efficient virus rescue using RNAs already containing such an RNA insert. This explains the high recombination incidence examined. Recently, similar events have also been shown for influenza virus [53].

BTV deletion mutants have inserted RNA sequences exclusively from viral origin and exclusively in the positive orientation. Generally, viral RNA synthesis of *Reoviridae* members is compartmentalized and synchronized. Plus strand RNA synthesis to generate mRNA occurs only in core particles, synthesis of minus strand RNA to form dsRNA occurs only after assembly in newly formed virus particles. Therefore, template switch for replicative recombination will occur between strands of viral origin and of the same polarity. On the other hand, the rescue of mutant ΔD(S2inverted) showed that inserts in the inverted orientation are not functional and nonviral RNA inserts are not beneficial for virus replication as was shown by rescue of mutant ΔD(S2delGFP).

The RNA sequences seem to be inserted at random positions in Seg-10. Also, the rescue of mutant ΔD(S2reposition) showed that the inserts are still functional at another location in Seg-10. This indicates that the inserts are independent of adjacent sequences and function as distinct *in cis* RNA elements.

Sequences of all found inserts were compared but no sequence or structure homologies were found. In a few occasions overlapping sequences or very similar inserts were independently found suggesting a preference of inserting these sequences.

Additional to encoding proteins, viral RNAs contain functions important for a variety of processes, such as transcription, replication and recruitment for packaging in the virus particle. RNA secondary structures and in particular pseudoknots are associated with a remarkable range of functions often involved in initiation of translation and ribosomal frame shifting, but could also be binding sites for proteins or single-stranded loops of RNA [54]. Kissing-loop interactions between viral segments by pseudoknots was already shown for other virus species [55]. More research on these RNA inserts is needed to unravel their precise role in virus replication. For these studies, the Seg-2 insert is very attractive due to its small size (67 bp) and predicted RNA pseudoknot structure.

Obviously, RNA inserts considerably enhance the efficiency of virus rescue using reverse genetics and are always found in deletion Seg-10 without expression of functional NS3. However, the mechanism in which these RNA sequences are involved is yet unknown.

One possibility is the recognition by NS2. NS2 is involved in the formation of VIBs [56], but also binds BTV-RNA. NS2 does recognize BTV RNA by the UTR’s, but also by yet unidentified RNA structures in ORFs [15], [16], [37].

The inserts can also be bound by VP6. It is suggested that VP6 binds to RNA for its helicase activity, but also plays a role in RNA packaging by a still unknown mechanism [57].

Another possibility for insert necessity, is that the optimal length of Seg-10 might be advantageous for the stability of a virus particle, since it is known that RNA can direct the assembly of the capsid and sometimes enhances capsid stability (reviewed by [58]). However, many small deletions in Seg-10 were less stable than the ΔD(S2del) with the smallest Seg-10 of only 320 base pairs in length, which was stable for more than 10 virus passages.

Genome segments of dsRNA in the virus particle are highly ordered. This ordering is partly due to interactions of dsRNA with VP3, but neighbouring RNA segments also seem to interact [13], [14]. The exact interactions in the virus particle are still unknown, but the RNA inserts could stabilize these interactions.

Although the found RNA inserts are needed for virus replication, their genetic stability is variable. A firstly generated virus variant with an insert in deletion Seg-10 can be overgrown by a newly arisen virus variant. After extensive passaging of mutant ΔD(S2delGFP), the original Seg-2 insert eventually changed into an insertion of Seg-6. It will be interesting to continue passaging of virus mutants in order to find the most optimal RNA sequence of deletion Seg-10 without expression of NS3/NS3a proteins. With the same aim, growth competition experiments between independently generated BTV mutants only differing in Seg-10 sequences could be performed.

Taken together, in addition to encoding proteins, RNA in BTV ORFs is also essential for virus replication itself. This system, in which RNA elements can be studied in virus replication without interference of translation, is a first step to elucidate the exact role and function of these RNA elements. The developed system with the protein-lacking genome segment 10 enables research on the role of RNA sequences in RNA replication, virus assembly, segment recognition and other processes in which RNA-RNA or protein-RNA interactions in the replication of dsRNA viruses are involved. Processes such as viral evolution and inter- and intragenic recombination can also be studied now.
